# Effects of Short-Term Physical Activity Interventions on Simple and Choice Response Times

**DOI:** 10.1155/2016/5613767

**Published:** 2016-04-13

**Authors:** Kevin Norton, Lynda Norton, Nicole Lewis

**Affiliations:** ^1^Faculty of Health Sciences, University of South Australia, Adelaide, SA 5001, Australia; ^2^Faculty of Medicine, Nursing and Health Sciences, Flinders University, Adelaide, SA 5042, Australia

## Abstract

*Objective*. Response time (RT) is important for health and human performance and provides insight into cognitive processes. It deteriorates with age, is associated with chronic physical activity (PA), and improves with PA interventions. We investigated associations between the amount and type of PA undertaken and the rate of change in RT for low-active adults across the age range 18–63 yr.* Methods*. Insufficiently active adults were assigned to either a walking (*n* = 263) or higher-intensity (*n* = 380) exercise program conducted over 40 days. Active controls were also recruited (*n* = 135). Simple response time (SRT) and choice response time (CRT) were measured before and after the intervention and at 3-, 6-, and 12-month follow-up.* Results*. SRT and CRT slowed across the age range; however, habitually active participants at baseline had significantly faster CRT (*p* < 0.05). The interventions increased weekly PA with corresponding increases in physical fitness. These changes were mirrored in faster CRT across the study for both intervention groups (*p* < 0.05). No changes were found for SRT.* Conclusions*. Both PA interventions resulted in improvements in CRT among adults starting from a low activity base. These improvements were relatively rapid and occurred in both interventions despite large differences in exercise volume, type, and intensity. There were no effects on SRT in either intervention.

## 1. Introduction

Response time (RT) is an important phenomenon in health and human performance. It is the time taken to physically react to a stimulus. Quantitatively it is the combination of processing time, or the time taken from the onset of a stimulus to the initiation of a volitional response, plus movement time [1]. In its most basic form it is called simple response time (SRT) and involves a single response to a signal.

The processing time combines the premotor period for the stimulus to reach the brain and the processing interval where activities such as recognition, association, coordination, inhibition, and decision-planning stages take place [2, 3]. These cognitive activities are especially important when there are multiple choices from which a potential response is made such as in choice response time (CRT) tasks [4]. Collectively, these (and other) more complex operations are often referred to as executive-control processes or executive functions [5].

Response time is important for health because it is associated with balance [6], the rate of voluntary stepping and mobility [7], probability of falls [8], and mortality [9, 10]. Slow RT is also related to driving errors such as collisions and traffic light violations in simulated driving tests [11]. For athletes involved in high-performance sport rapid anticipation, decision-making and movement speeds are critical for success and have been shown to discriminate the very best athletes from others [12].

It has been known for over a century that RT provides an insight into cognitive function [13]. This is because the central processing component of RT can be easily demonstrated and manipulated by varying the type and nature of the stimulus and response required. For example, simple RT is shorter than a recognition RT, and CRT takes even longer because the subject must choose a specific response corresponding to the stimulus. Declines in cognitive performance are associated with aging, brain-injury, and other neurodegenerative pathologies [14]. A meta-analysis of age and cognitive function has found that around 75% of the age-related variance in several cognitive variables was also common with RT [15]. Physical activity (PA), however, has been shown to be associated with improved cognitive function in cross-sectional [16, 17], experimental [18–20], and longitudinal studies [21, 22]. Improvements have also been found in balance and frequency of falls in physically active intervention subjects versus less active controls [23]. A meta-analysis of aerobic exercise interventions and neurocognitive performance found modest improvements in processing speed and executive function [4]. Despite these findings there are still many unanswered questions concerning the relationships between PA and cognitive function and larger trials with longer follow-up are required [2, 4, 24]. In particular, as populations in many parts of the world continue to age and PA patterns remain low [25] there is a need to better understand the interaction of the amount and type of activity for optimal brain function and the time course for changes in cognitive function to take place using exercise interventions.

The study aims were to investigate the (1) relationships between RT and age in large cross-sectional cohorts of insufficiently active and regularly active participants and (2) effects of two types of PA interventions on RT in previously insufficiently active adults.

## 2. Materials and Methods

The methods used in this study are reported in more detail in Norton et al. [26] and in a paper reporting a range of other health and fitness changes as part of a large PA intervention [27, 28]. A brief overview is provided here.

### 2.1. Subjects

Ethics approval for this study was obtained from the institution's Research Ethics Committee. Adults aged 18–63 years were recruited following email advertising sent throughout a university, a tertiary hospital, and several government departments. Potential subjects were invited to attend the Exercise Research Laboratory at the University to complete informed consent and a questionnaire on their PA patterns covering the previous week [27, 28]. Those undertaking insufficient levels of PA for health benefits (InSA; <150 min/wk) were invited as intervention participants while the active subjects (SA; ≥150 min/wk) could volunteer to act as controls if this activity pattern was reported to be consistent over the previous 12 months. There were 312 insufficiently active subjects who were randomly assigned to one of two types of interventions: (1) a limited contact (information-oriented) pedometer-based strategy (*n* = 157) or (2) an active (instructor led) group-based strategy (*n* = 155) [26]. There were additional 106 subjects who could not attend the structured group intervention classes (for various reasons) but were otherwise willing to participate in the pedometer arm of the study giving a total of 263 pedometer participants. Further 225 nonrandomised participants, primarily from a university population, were recruited and undertook the group-based intervention giving a total of 380 group participants. There were no differences in the gender or physical activity patterns between the randomised versus nonrandomised subjects either before or after intervention and the additional nonrandomised subjects are, therefore, included in the present study. All intervention subjects were tested before intervention for baseline measures of RT. The intervention phase ran for 40 days at which time a postintervention test was conducted. Subjects then undertook follow-up testing at three, six, and 12 months after intervention with the exception of the additional group subjects who were only involved in the intervention phase of the study. The active control group (*n* = 135) was tested at baseline and six- and 12-month follow-up.

### 2.2. Interventions

Subjects in the pedometer intervention were equipped with a pedometer (Yamax Digiwalker SW-700) and a diary. They were instructed on how and when to wear the pedometer and were asked to record their step count at the end of each day. In the first week of the intervention subjects were instructed to achieve at least 5,000 steps each day, gradually increasing to 10,000 steps each day by the last week of the intervention. Weekly emails were sent to all pedometer subjects outlining the step count goal for the week and tips to increase walking activity [26].

The group intervention combined a number of elements that have previously been shown to be important for long-term behavioural change. Subjects attended the university three times each week for group activities led by instructors. Subjects participated in activity of their own choice on alternate days. Heart rate monitors (Polar S610, Polar Electro Oy, Finland) were worn during all activity sessions and subjects completed a daily diary of physical activity including type, duration, intensity, and rating of perceived exertion (RPE). Researchers downloaded HR monitors weekly to record exercise duration, %  HR_max_, and estimated energy expenditure. Group sessions were designed to have participants expend approximately 800 kJ/session in the first week and increase by about 200 kJ/session in each subsequent week. Activity sessions lasted 60 min with a core of about 40 min between approximately 75 and 85% of estimated HR_max_.

Physical activity patterns, response time, and cardiorespiratory fitness were assessed for all participants at each test session. Minutes of PA during the previous week were quantified using the Active Australia Survey [27, 28] and no weighting adjustments were made for vigorous PA. Cardiorespiratory fitness was measured using submaximal assessment protocols on a bicycle ergometer [29] to predict maximal aerobic capacity [26].

### 2.3. Measurement of RT

On the test day subjects were instructed on how to use the RT equipment and the research staff made demonstrations of both SRT and CRT testing protocols. Subjects were then given several practice trials on the RT equipment to ensure they were familiar with the procedures before recordings were made. The SRT test involved the following sequence: subjects used the index finger of their dominant hand while seated in front of a keypad and computer screen; an audible “beep” was provided by the computer to indicate each trial was about to begin; a random fore period of between 1 and 3 seconds was used followed by a light switch being illuminated; subjects pressed a key as quickly as possible; and response time was recorded electronically to the nearest millisecond. The CRT test involved the same apparatus. Subjects sat in front of a keypad with four buttons and a random fore period was used together with a random illumination of one of four lights. Subjects pressed a button corresponding to the illuminated light as quickly as possible. In both tests ten trials were performed, the shortest and longest times were discarded, and the mean of the remaining eight trials was used in further analyses.

### 2.4. Statistical Analysis

Linear regression was used to determine the association between age and RT. Differences in intercepts and slopes between regression lines were calculated using *t*-tests. A longitudinal mixed model with random effects (REMM) imputation method was used to assess changes in RT over time [30]. This addresses the problem of missing data by using a model that estimates the trend shown by the subject over the available data and augments this by the trend from the whole sample [31]. This method is an intention to treat process and all data points are included in the models. The three groups were initially analysed separately and then all intervention subjects were combined for a separate REMM analysis. A significant group *x* time interaction effect indicated a significant difference in RT among (between) the groups across the study period. Alpha was set at 0.05.

## 3. Results

Descriptive details of the subjects are provided in Table 1. The mean age of the group intervention subjects was less than the pedometer and control groups (*p* < 0.0001). There were significantly more females than males who volunteered for this study but this was consistent across both intervention and control arms. The increased total PA levels were dramatic for both intervention arms across the 40 days of activity. The control subjects were highly active and proved to be a stable reference group across the duration of the study.

**Figure 1:**
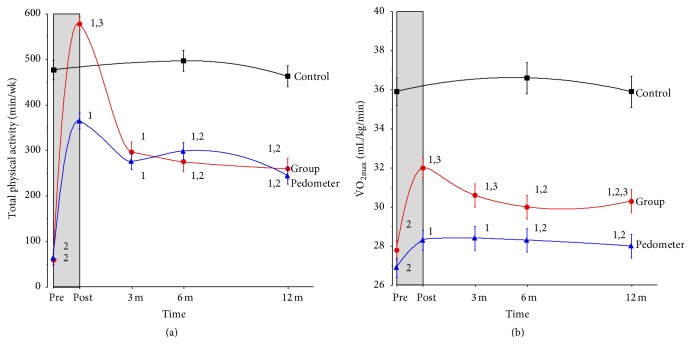
REMM results for PA and VO_2max_ across the study. The grey area represents the intervention phase. Values are mean ± SE. 1 = difference versus preintervention (within group); 2 = difference versus control; 3 = difference versus pedometer. Interpolation lines are computer-generated.

As expected, PA patterns decreased over the follow-up period although significantly higher PA levels were found for both intervention arms at 12 months after intervention relative to the low preintervention levels. The levels of vigorous activity during the interventions (minutes per week) increased from medians (mean ± SD) of 0 (6 ± 14) to 40 (74 ± 105) and from 0 (7 ± 15) to 240 (265 ± 164) in the pedometer and group arms, respectively. The elevated PA patterns reported by the subjects were supported by superior measures of cardiorespiratory fitness. VO_2max_ (mean ± SD in mL/kg/min) increased across the 40-day intervention from 27.2 ± 6.9 to 28.6 ± 8.2 and from 27.7 ± 6.9 to 32.4 ± 7.8, in the pedometer (*n* = 209; *p* = 0.0003) and group (*n* = 283; *p* < 0.0001) intervention arms, respectively. Using either REMM or per protocol analysis showed the control subjects were a very stable reference group for both PA patterns and cardiorespiratory fitness (Figure 1). For example, per protocol results showed the controls who completed both follow-up tests (*n* = 96) had baseline values of 38.1 ± 11.2 mL/kg/min and these were unchanged across the study (38.8 ± 10.8 and 38.3 ± 11.5 at the six- and 12-month tests, resp.; *p* = 0.53).

### 3.1. Response Time

**Figure 2:**
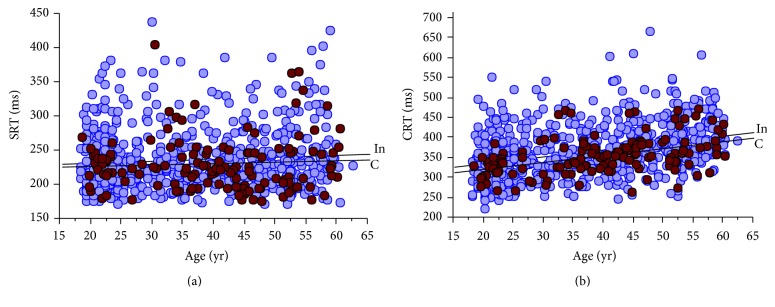
The change in RT with age. (a) shows there was no association in SRT with age for the control participants (controls (C) = darker circles; *n* = 135; *y* = 221.4 + 0.20*∗x*; *p* = 0.478) while there was a weak positive association for the intervention participants (intervention (In); *n* = 641; *y* = 224.9 + 0.28*∗x*; *p* = 0.038). (b) shows there were significant positive associations in CRT with age for both the control (controls (C) = darker circles; *n* = 135; *y* = 285.4 + 1.74*∗x*; *p* < 0.0001) and intervention participants (intervention (In); *n* = 643; *y* = 301.7 + 1.61*∗x*; *p* < 0.0001).

Figure 2 shows the relationships of RT versus age and between participants who were active versus insufficiently active prior to the intervention. Regression analysis of all preintervention RT measures (using all subjects, *n* = 776) showed both SRT (*y* = 224.8 + 0.25*∗x*; *p* = 0.038) and CRT (*y* = 300.1 + 1.60*∗x*; *p* < 0.0001) increase across the age span. SRT deteriorated at a rate of about 1.1% per decade while CRT slowed at about 5.3% per decade. Comparison of the SRT versus age regression lines between the intervention and control groups showed no differences for slope (*t* = 0.17; *p* = 0.87) nor intercept (*t* = 0.81; *p* > 0.05). There was no difference in the regression slopes for CRT (*t* = 0.37; *p* = 0.71); however, there was a significant difference between intercepts (*t* = 1.79; *p* < 0.05) where the controls had faster CRT that remained consistent across the age span.

**Figure 3:**
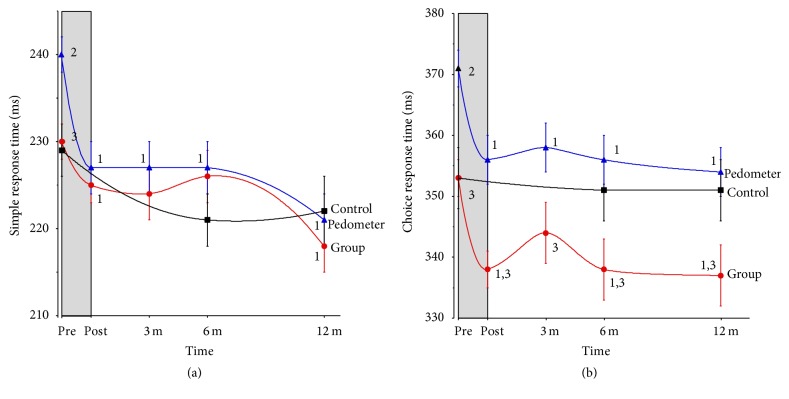
REMM results for the change in RT over the duration of the study. The grey area represents the intervention phase. Values are mean ± SE. 1 = difference versus preintervention (within group); 2 = difference versus control; 3 = difference versus pedometer. Interpolation lines are computer-generated.

REMM analysis indicated there was no difference in the patterns of change in SRT among the three groups across the 12-month study period.  Figure 3(a) shows there was a similar learning effect for all groups. Similarly, REMM results for the combined intervention participants versus controls showed no differences in group *x* time for SRT (*p* = 0.168).  Figure 3(b) shows CRT decreased significantly for both the group (*p* = 0.013) and pedometer (*p* < 0.0001) participants with no change for the control group (*p* = 0.056). There was no difference in regression slopes between the group and pedometer participants (*p* = 0.923). REMM results for the combined intervention participants versus controls showed a difference in group *x* time for CRT (*p* = 0.028). Overall, there was a medium effect size (0.55) in CRT for the intervention group from baseline to end (95% CI = 0.44–0.66), while the effect size for the control group (0.09) was not significant (−0.15–0.33).

## 4. Discussion

This is one of the largest studies reporting changes in RT across a range of ages following PA interventions [2, 4, 32]. Preintervention analysis using all participants showed both SRT and CRT slowed across the age range. However, participants who were habitually active at baseline had significantly faster CRT across the age range. No differences were found between rates of decline in either SRT or CRT between active and insufficiently active participants. The 40-day intensive PA interventions resulted in average increases in weekly activity patterns of about 6–9-fold for the pedometer and group-based programs, respectively. Correspondingly, the predicted VO_2max_ values also increased, averaging approximately 5 and 17% in these two intervention groups, respectively. These results confirmed relatively rapid physiological responses to the increased physical loads. In turn, using REMM analysis and with adjustments for learning effects, the PA interventions improved CRT by an average of approximately 3.8 and 3.4% for the pedometer and group intervention participants, respectively. The PA interventions had no significant effect on SRT.

Previous research has found a relatively consistent pattern of slower RT versus age in both cross-sectional [13, 33, 34] and longitudinal studies [33, 35, 36], and almost all report increasing intra-individual RT variability with age [13]. The results are often curvilinear when the age range extends beyond about 50 years for SRT whereas increases have been shown to accelerate for CRT much earlier [13]. In the present study, however, nonlinear regression analysis was not significant for SRT versus age (*p* = 0.064 for a second-order polynomial). It was also not different from linear regression results for CRT where correlation coefficients were *r* = 0.361 and 0.360 with identical RMS residuals (0.053) for both linear and nonlinear regression analysis, respectively. Our relatively narrow age range of 18–63 yr may have accounted for the linear pattern of RT versus age. In a large cross-sectional study of over 7,000 participants, and using tests comparable to those in the present study, the rates of change in RT across the age range 20–60 years increased at about 2.2% for SRT and 5.1% per decade for a four-choice CRT test [13]. A study by Fozard and colleagues [33] of 1,265 participants also showed linear changes in SRT and CRT equivalent to increases of about 2.2% and 4.9% per decade, respectively, despite an age range from 17 to 96 years. The present study showed similar changes for SRT (1.1%) and CRT (5.3%) versus age. The significantly lower CRT for the physically active participants relative to those who were insufficiently active at preintervention across the age range is important and may represent effects of longer-term PA on RT. The results are also consistent with others who have reported physically active people performing better on cognitive tests including RT [17, 18, 20] and have less brain tissue loss with aging [2]. Given that CRT declines early in adulthood and that being active helps to attenuate these declines, it is important that people remain active throughout life for both cognitive and physical health. Physical activity has been shown to improve and maintain brain function, particularly attention, processing speed and memory [4, 37, 38], and motor performance [9, 39] and these are critical in our aging population for activities such as driving and independent living and as predictors of dementia [40], falls [41], and mortality [9]. Overall, the majority of observational studies have shown a positive relationship between PA or cardiovascular fitness and cognitive ability [16, 38, 42, 43] and human PA intervention studies have been associated with improvements in brain systems and performance [4, 32, 44] and increases in brain matter [45].

It is because of the enormous range of health benefits, including cognitive-related improvements, that health promotion campaigns focus on increasing population levels of PA [46, 47]. While the amount and type of PA required for cardiovascular health are relatively well established [48], less is known about the dose-response relationships for optimal brain health [32, 37, 38]. The present study investigated the effects of two types of PA interventions on RT in previously insufficiently active adults. The pedometer intervention participants increased PA across the 40-day intervention from a median of 65 to 365 min/wk while the group-led participants increased substantially more from 60 to 510 min/wk. Despite such impressive increases in PA and the corresponding increases in cardiorespiratory fitness (Figure 1), there were no changes in SRT in either intervention arm. The lack of change in SRT has also been reported in many other studies [18, 23, 32, 49].

On the other hand, CRT showed significant improvements across the 40-day intervention phase of the study in a manner that essentially mirrored the changes in PA and fitness (Figures 1 and 3). Even though there were large differences in PA patterns between the intervention groups, the CRT changes were similar. The differences in PA volume during the intervention showed they were almost exclusively due to the higher levels of vigorous PA undertaken by the group participants (median of 240 versus 40 min/wk for the pedometer participants). This suggests that either the moderate intensity activity, lower levels of vigorous PA, or the combination was sufficient to enhance cognitive performance and discriminatory speed as measured in the CRT task for previously insufficiently inactive adults. It is not possible to further refine the specific thresholds of moderate or vigorous PA for these improvements. However, REMM analysis showed the improved CRT persisted even though the longer-term patterns of PA decreased. For example, at 12 months the median levels of total PA were 200 and 180 min/wk (median vigorous PA levels were 30 and 60 min/wk) for the pedometer and group intervention arms, respectively. The results, therefore, approximate the broad recommendations of at least 150 min/wk of PA to achieve health benefits [48]. A meta-analysis using 24 studies found an effect size for aerobic exercise and subsequent changes in attention and processing speed of 0.158 (CI = 0.055–0.260) and greater improvements for combined (aerobic and strength-based training) versus aerobic only interventions [4]. Our group intervention involved a range of activities including circuit and resistance training that would be categorised as a “combined” trial [26]. Since there were equally impressive improvements in CRT in both intervention arms, the combined activities made little difference in RT changes relative to the walking-based pedometer program.

## 5. Limitations

There was a significant age difference between the intervention groups. This may have impacted the potential to respond to a PA intervention. For example, the younger group intervention participants exhibited faster CRT before intervention and this might have limited their capacity to improve despite their impressive gains in PA. In other words, the additional vigorous and total PA for the group participants may have resulted in even greater changes if they were older and had slower CRT before intervention. The PA patterns are based on questionnaire responses and are therefore subject to social desirability bias. However, the measured cardiorespiratory fitness changes support the PA data as other reported health risk factor changes do [27, 28].

## 6. Conclusions

Our cross-sectional analysis showed age-related changes in both RT measures. CRT increased at almost five times the rate of SRT between 18 and 63 yr. The active control group had significantly faster CRT across the age range studied compared to the insufficiently active participants suggesting enhanced processing speed may result from chronic PA habits. This is supported in the present study by the findings that both types of PA interventions resulted in improvements in CRT among adults starting from a low activity base. These improvements were relatively rapid and occurred in both PA programs despite differences in exercise volume, type, and intensity. There were no effects on SRT in either intervention arm relative to controls.

## Figures and Tables

**Table 1:** Descriptive data for the subjects. Physical activity (PA) data are total median (mean ± SD) minutes per week using REMM analysis. There were two participants from the group intervention arm who had missing preintervention RT data.

	Group	Pedometer	All intervention	Controls
*n*	380	263	643	135
Age at enrolment; mean ± SD (yr)	33.6 (12)	40.0 (13)	36.7 (13)	39.1 (12)
Gender (% F)	73	76	74	71
Preintervention PA (min/wk)	60 (60 ± 41)	60 (65 ± 42)	60 (62 ± 41)	405 (477 ± 270)
Postintervention PA (min/wk)	510 (556 ± 296)	270 (365 ± 308)	420 (478 ± 315)	
3-month PA (min/wk)	265 (309 ± 236)	220 (286 ± 238)	240 (293 ± 237)	
6-month PA (min/wk)	240 (284 ± 272)	240 (301 ± 275)	240 (294 ± 273)	395 (505 ± 388)
12-month PA (min/wk)	180 (270 ± 224)	200 (248 ± 211)	195 (256 ± 216)	365 (468 ± 366)

## Acronyms

CI: Confidence interval
CRT: Choice response time
HR: Heart rate
HR_max_: Maximum heart rate
InSA: Insufficiently active
PA: Physical activity
REMM: Random effects mixed modelling
RPE: Rating of perceived exertion
RT: Response time
SRT: Simple response time
SA: Sufficiently active
SD: Standard deviation
SE: Standard error
VO_2max_: Maximal oxygen uptake.
